# Designing and Characterization of a Novel Delivery System for Improved Cellular Uptake by Brain Using Dendronised Apo-E-Derived Peptide

**DOI:** 10.3389/fbioe.2019.00049

**Published:** 2019-03-26

**Authors:** Shafq Al-azzawi, Dhafir Masheta, Anna Guildford, Gary Phillips, Matteo Santin

**Affiliations:** Centre for Regenerative Medicine and Devices, School of Pharmacy and Biomolecular Sciences, University of Brighton, Brighton, United Kingdom

**Keywords:** neurodegenerative disease, blood-brain barrier, dendrimers, drug delivery system, cellular uptake

## Abstract

Neurodegenerative diseases (ND) are characterized by the progressive loss of neuronal structure or function mostly associated with neuronal death. The presence of the blood–brain barrier (BBB) is considered the main obstacle that prevents the penetration of almost all drugs rendering the diseases untreatable. Currently, one of the most promising approaches for drug delivery to the brain is by employing endogenous transcytosis to improve endothelial cell uptake. This study aimed to exploit this potential route of enhanced drug uptake through the design and characterization of low generations lysine dendrons with further functionalization of dendron with ApoE-derived peptide (AEP) ligand to improve cellular uptake and targeting of delivery to the brain. Dendrons and peptide were synthesized using solid phase peptide chemistry and the products were characterized by mass spectrometry and high performance liquid chromatography which confirmed the successful synthesis of dendrons and functionalization with the AEP. Cell viability and lactate dehydrogenase release were conducted to study the cytotoxicity of the materials against an immortalized brain endothelial cell line (bEnd.3) which demonstrated that no toxicity was seen at the concentration range used (up to 400 μM) for up to 48 h incubation. Cellular uptake of the synthesized molecules was examined using confocal microscopy and flow cytometer which clearly showed the cellular uptake of the dendronised carrier systems and that the highest percentage of cellular uptake was achieved with the AEP-functionalized dendron. This study has therefore demonstrated the successful synthesis of dendronised carrier systems with the potential to act as carriers for improved delivery and targeting the brain.

## Introduction

The early diagnosis and efficacious treatment of neurodegenerative diseases (NDs), including Alzheimer's disease, Parkinson's disease and multiple sclerosis, are significantly compromised by the presence of the blood–brain barrier (BBB), a membrane made of astrocytes and endothelial cells connected by tight junctions that prevents the sufficient penetration of almost all drugs, genes and imaging agents to the brain (Re et al., 2012; Wyss, 2016). Several approaches have been considered to penetrate the BBB including temporary disruption or opening of the BBB by chemical methods. However, these strategies are limited by the lack of selectivity that allows the penetration of other unwanted substances in the brain (Banks, 2012). The chemical modification of drugs with functional groups facilitating cell internalization have also been proposed (Wermuth et al., 2015), but their applicability is limited to the suitability of the drug properties for chemical derivatisation and to the alteration of their therapeutic efficacy (Chen and Liu, 2012).

Transport-vector strategies have recently been developed to deliver bioactive molecules with low BBB permeability to the brain (Kumar et al., 2015). These strategies capitalize either on the temporary destabilization of the tight junction sealing the extracellular endothelial space or on the internalization of macromolecules by the endothelial cells (Khawli and Prabhu, 2013). In the latter approach, the design of the transport-vector is based on the exploitation of the two main transcytosis pathways: (i) the adsorptive-mediated transcytosis (AMT) relying on the ability of hydrophobic molecules to penetrate the phospholipidic plasmalemma (Herve et al., 2008) and (ii) the receptor-mediated transcytosis (RMT) exploiting the biospecific recognition of ligands by cell receptors involved in the transport of molecules essential to the brain physiology (Chen and Liu, 2012).

The use of these carriers is one of the most promising as it has the potential of combining effective transport to the lack of drawbacks such as disruption of the BBB integrity and cell toxicity (Khawli and Prabhu, 2013). Therefore, these concepts have been applied to a range of widely recognized drug delivery system; these include micelles, vesicle, liposomes, polymers, dendrimers and nanoparticles (Sahoo et al., 2007). It is postulated that, while these drug delivery systems improve drug solubility, payload (Tiwari et al., 2012) and dosage (Sahoo et al., 2007), their coupling with molecules able to exploit AMT or RMT will enhance their BBB penetration. The combination of these properties will provide a carrier platform suitable for the delivery of various types of drugs or diagnostics (Re et al., 2012).

It is understood that the integration of an AMT- or RMT-specific molecule in the carrier needs to be conceived to enhance its presentation to the cell membrane and receptors while maintaining the ability to form a stable complex with the transported drug.

Dendrimers are hyperbranched polymeric macromolecules that can be synthesized from different monomers to obtain structures of well-defined order, size and polydispersity index (Heather et al., 2011). The high density of their terminal functional groups is one of the key properties as it provides multiple attachment sites for the complexation of drugs or other bioactive molecules. In addition, dendrimers are metabolized by cellular hydrolytic enzymes and completely biodegraded into their non-toxic building monomers (Sadekar et al., 2013). Because of these properties, dendrimers have been proposed as nanocarriers with a high therapeutic potential (Khawli and Prabhu, 2013); they have efficiently been used in many pharmaceutical and personal care applications (Sahoo et al., 2007) and for applications in cancer therapy and imaging (Lee and Nan, 2012). Polyamidoamine (PAMAM) dendrimers conjugated with the anticancer drug, camptothecin, have been shown to enhance the drug solubility and to increase its bioavailability at the target tissue (Sadekar et al., 2013).

Noticeably, it has also been observed that PAMAM dendrimers complexed with non-steroidal anti-inflammatory drugs such as ketoprofen and indomethacin improved the drug permeation through the skin (Cheng et al., 2007) and that in the case of oral drug delivery they can cross cell membranes (Patri and Simanek, 2012). Likewise, it was also found that dendrimer-ibuprofen complexes were able to enter the lung cells more rapidly when compared with the free drug (Kolhe et al., 2003) and that the conjugation of doxorubicin to a polyethyleneglycol (PEG) dendrimer enhanced cellular uptake by the brain and reduced tumor volume of glioma spheroids (Li et al., 2012; Xu et al., 2014).

However, all these drug carrier systems were not specifically designed to guarantee enhanced interaction with the cell plasmalemma or bio-specificity. Bio-specificity has recently been pursued for a number of applications by the introduction of a novel class of dendrimer derivatives, the dendrons. These are macromolecules with a tree-like structure able to present different functionalities at their molecular root and at their uppermost branching terminals (Meikle et al., 2011). The dual functionality enables the carriers to form stable and specific complexes with bioactive molecules (Meikle et al., 2016; Perugini et al., 2018) and/or magnetic resonance contrast agents (i.e., magnetic nanoparticles) while enhancing cell internalization processes (Maggio et al., 2016). These specific designs of dendrons are made possible by the solid phase synthesis of poly(epsilon-Lysine) branched peptides that ensures the controlled presentation of the desired functionalities in terms of both orientation and spacing (Meikle et al., 2011).

The aim of this work was to develop AMT- and RMT-competent poly(epsilon-Lysine) dendrons as a carrier platform for the delivery of bioactive molecules and contrast agents to the brain by enhanced BBB endothelial cells targeting and internalization.

In the case of AMT-competent dendrons a hydrophobic amino acid, the phenylalanine, was integrated at the root of the dendron in the view of enhancing the hydrophobic interaction of the carrier with the phospholipidic membrane. Whereas in the case of RMT-competent dendrons, the root of the dendron integrated a peptide sequence (LRKLRKRLLR) (Sauer et al., 2005; Gobbi et al., 2010). This peptide sequence has been previously identified as an analogue of the apolipoprotein E (ApoE), a protein recognized by the low density lipoprotein receptor (LDLr) that is a receptor involved in the transcytosis of molecules across the BBB (Xiao and Gan, 2013; Molino et al., 2017).

The present work focussed on achieving a high level of purity of the synthesized macromolecules and on the assessment of the impact of their size and AMT or RMT functionality on the efficiency of BBB endothelial cells internalization process.

## Materials and Methods

### Synthesis of Delivery Systems

**Figure 1:**
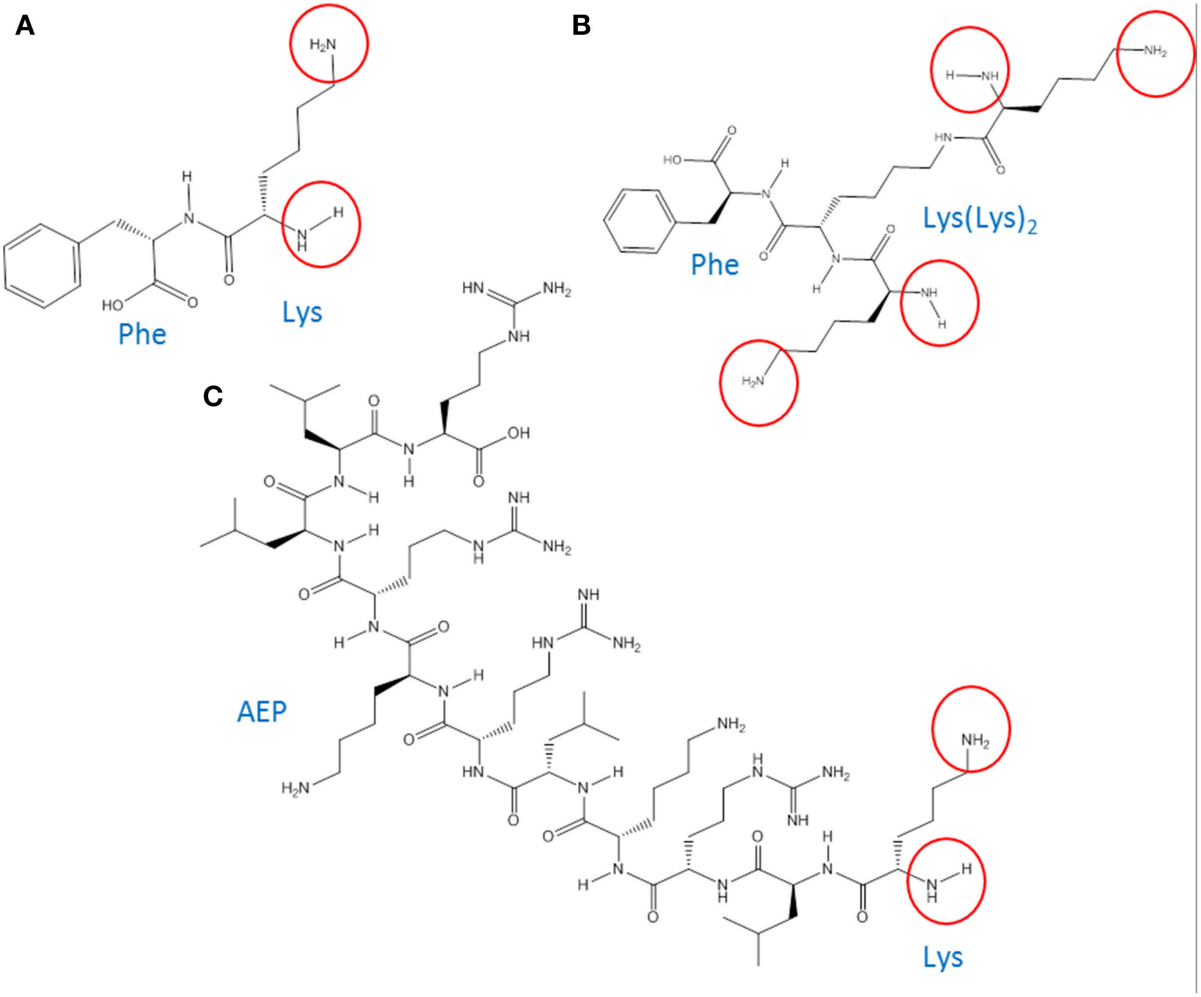
Chemical structure of **(A)** G0-dendron (chemical formula: C_15_H_23_N_3_O_3_, molecular weight (MW): 293.3 Da). **(B)** G1-dendron (chemical formula: C_27_H_47_N_7_O_5_, MW: 549.7 Da). **(C)** AEP-dendron (chemical formula: C_66_H_130_N_26_O_12_, MW: 1479.9 Da). The encircled groups represent the free amino terminal groups.

**Figure 2:**
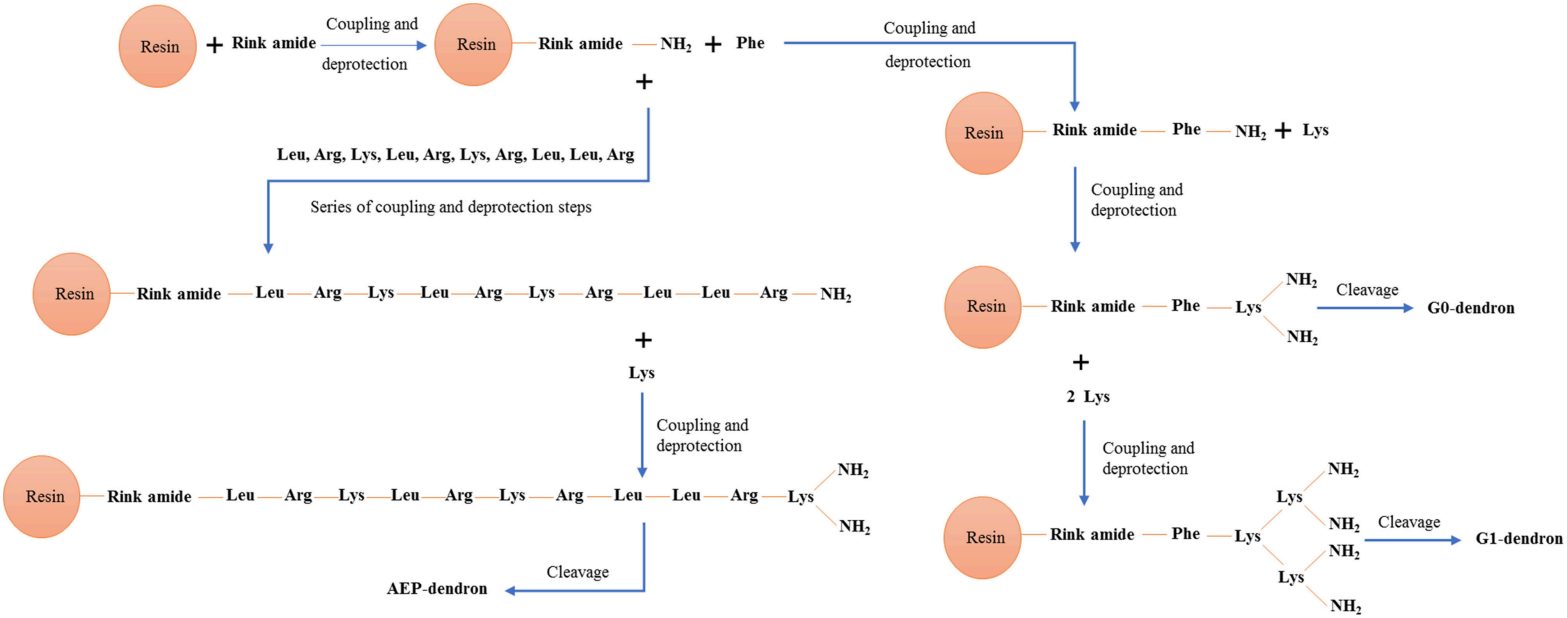
Schematic diagram showing SPPS chemistry including coupling and deprotection of amino acids monomers for synthesis of G0-, G1-, and AEP-dendron.

Poly(epsilon-Lysine) dendrons with branching generation 0 (G0, two exposed amino groups) and G1 (4 exposed amino groups) were synthesized using a high-yield solid phase peptide synthesis (SPPS) (Shin et al., 2005; Made et al., 2014) based on the use of a microwave system (Biotage Initiator, UK). The synthesis of AMT-competent dendrons included the integration of a Phenlyalanine monomer at its molecular root (Figures 1A,B). In the case of the G0 dendron an alternative RMT-competent formulation was synthesized by integrating the ApoE-mimicking sequence, LRKLRKRLLR in the dendron root (Figure 1C). Both types of dendrons were assembled on a Tentagel NH_2_ resin previously coupled with a Rink-amide-linker susceptible to cleavage and necessary for the liberation of the dendron at the end of the synthesis (Meikle et al., 2011). The dendron assembly on the solid phase support was performed through different cycles each one using 0.4 mmol of Fmoc-protected amino acids (Novabiochem, UK) (Figure 2). In the case of the AMT-competent dendron the root was achieved by the grafting of a Fmoc-Phe-OH followed by the assembling of Fmoc-lys(Fmoc)-OH in cycles necessary for either a G0 and G1 branching. Whereas, a sequence of Fmoc-Leu-OH, Fmoc-Arg-OH, Fmoc-Lys(Boc)-OH, Fmoc-Leu-OH, Fmoc-Arg-OH, Fmoc-Lys(Boc)-OH, Fmoc-Arg-OH, Fmoc-Leu-OH, Fmoc-Leu-OH, Fmoc-Arg-OH consecutively were used for the synthesis of the APE linear peptide at the root of the RMT-competent dendron. The synthesis protocol included coupling of the Fmoc amino acid, its deprotection from the Fmoc group and cleavage of the final product from the resin as previously described (Meikle et al., 2011; Al-azzawi, 2017). The cleaved mixture was washed, filtered and collected in chilled diethylether. After a series of centrifugation and washing steps with diethyl ether, the precipitated peptides were collected and freeze dried. In order to remove any impurities and undesired by-products, a Zeba spin desalting column (Fisher scientific, UK) was used and the final pure products were used for characterization and *in vitro* cell experiments.

### Characterization of Dendronised Delivery Systems by Mass Spectrometry (MS)

The AMT-competent G0- and G1- dendrons as well as the RMT-competent AEP-dendron were characterized by electrospray/ionization-time of flight (ESI-TOF MS) (Bruker Daltonics, UK) at high voltage (4 kV). The samples were dissolved in methanol, then filtered using a 0.22 μm filter and injected into the spectrometer. In electrospray/ionization mode, sample mass (m/z) gave rise to multiple charged ions labeled with a number of charges (n) as (MW+nH)n+, where H is the mass of a proton (1.008 Da).

### Characterization of Dendronised Delivery Systems by High Performance Liquid Chromatography (HPLC)

Each sample was dissolved in methanol and filtered with a 0.22 μm filter before analysis. The analytical method was performed using HPLC, diode array detector (Agilent technology/1260 infinity, UK) with a Luna hydrophobic C18 column (150^*^4.6 mm) and a UV-detection wavelength of 223 nm, the optimum wavelength for these peptide dendrons (Al-azzawi, 2017). The HPLC mobile phase consists of water/acetonitrile in which the gradient of eluent was run from 75:25 to 25:75 water: acetonitrile over 20 min.

### Preparation of bEnd.3 Cell Line

The immortalized brain endothelial cell line, bEnd.3 (ATCC-CRL-2299), and Dulbecco's modified eagle's medium (DMEM) were obtained from ATCC (USA). The bEnd.3 cells were cultured, according to ATCC-product sheet instruction, in DMEM high glucose medium with L-pyruvate, containing 10 % (v/v) fetal bovine serum and 1% (v/v) of 500 U/ml Penicillin/Streptomycin (Gibco, Germany). Cells were seeded at a density of 5 × 10^4^ cells per cm^2^ in 24-well plates then incubated at 37°C and 5% CO_2_ and the culture media was replaced every 3 days.

### Cytotoxicity Assays

Experiments were performed when cells reached confluence. A range of concentrations (25, 50, 100, 150, 200, 300, and 400 μM) of each AMT-competent G0-, G1-dendron, and RMT-competent AEP-dendron were used in each experiment.

The MTT [(4,5-Dimethylthiazol-2-yl)-3,5-diphenylformazan thiazolyl blue tetrazolium] (Sigma Aldrich, UK) assay was used to measure cell viability (Mosmann, 1983). After 24 and 48 h treatment exposure, the MTT assay was conducted and the absorbance was measured at a wavelength of 540 nm by spectrophotometry (Thermo Multiskan Ascent, UK). Readings of 6 replicates were expressed as percentage of the untreated control cells.

Lactate dehydrogenase (LDH) was measured using Promega CytoTox96® non-radioactive cytotoxicity assay kit (UK) after 24 and 48 h incubation. Absorbance was read spectrophotometrically at 492 nm and converted to a percentage of the total LDH released from the positive control (cells subjected to complete lysis) for 6 replicates.

### Cellular Uptake Studies

#### Cellular Uptake Examination by Laser Scanning Confocal Microscopy (LSCM)

To study the cellular uptake of the designed delivery systems, the AMT-competent G0- and G1-dendrons, and the RMT-competent AEP-dendron were labeled with fluorescein-5-isothiocyanate (FITC) (Sigma Aldrich, UK). The labeling was performed by dark covalent coupling reaction with FITC solution with 2 molar excess for each terminal amino group. The products were washed with dichloromethane, methanol and diethylether (Fisher scientific, UK), consecutively to remove any unreacted substances.

The bEnd.3 cells were treated at confluence with FITC-labeled products dissolved in DMEM and incubated for 1 h (the appropriate incubation time for dendrons with this type of cell line). The cells were then fixed with 3.7% (v/v) formalin, and analyzed by LSCM (Leica TCS SP5, UK), using a 488-visible laser source.

#### Cellular Uptake Evaluation by Flow Cytometer

The bEnd.3 cells were cultured and treated at confluence with 100 μL FITC-labeled products dissolved in DMEM for 1 h. The cells were washed with phosphate buffer solution twice and trypsinised and then harvested to be analyzed using BD C6 sampler flow cytometer (BD Accuri C6, Bioscience, UK). Samples of cells without treatment were also included as a control, and the analysis was carried out through the FL1-H channel for FITC detection.

### Statistical Analysis

Mean values were calculated for the number of readings (*n* = 6) in each experiment and the error bars refer to the standard deviation (SD). Results were statistically analyzed using one-way ANOVA with Tukey's tests. Significant differences were identified by a *P*-value < 0.05.

## Results

### Characterization by Mass Spectrometry

**Figure 3:**
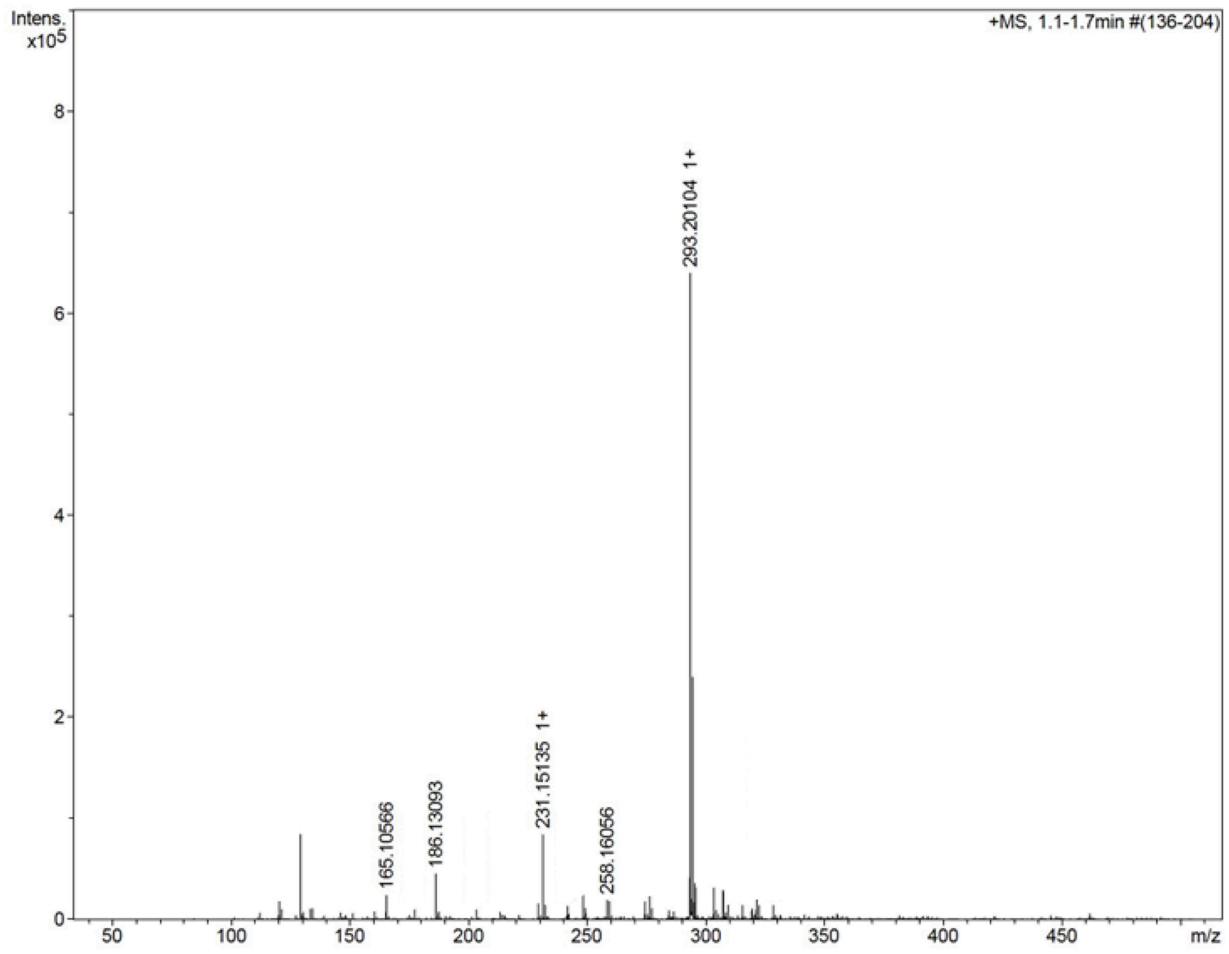
Mass spectrum of G0-dendron showing the main peak of 293.2 representing the exact MW of product.

**Figure 4:**
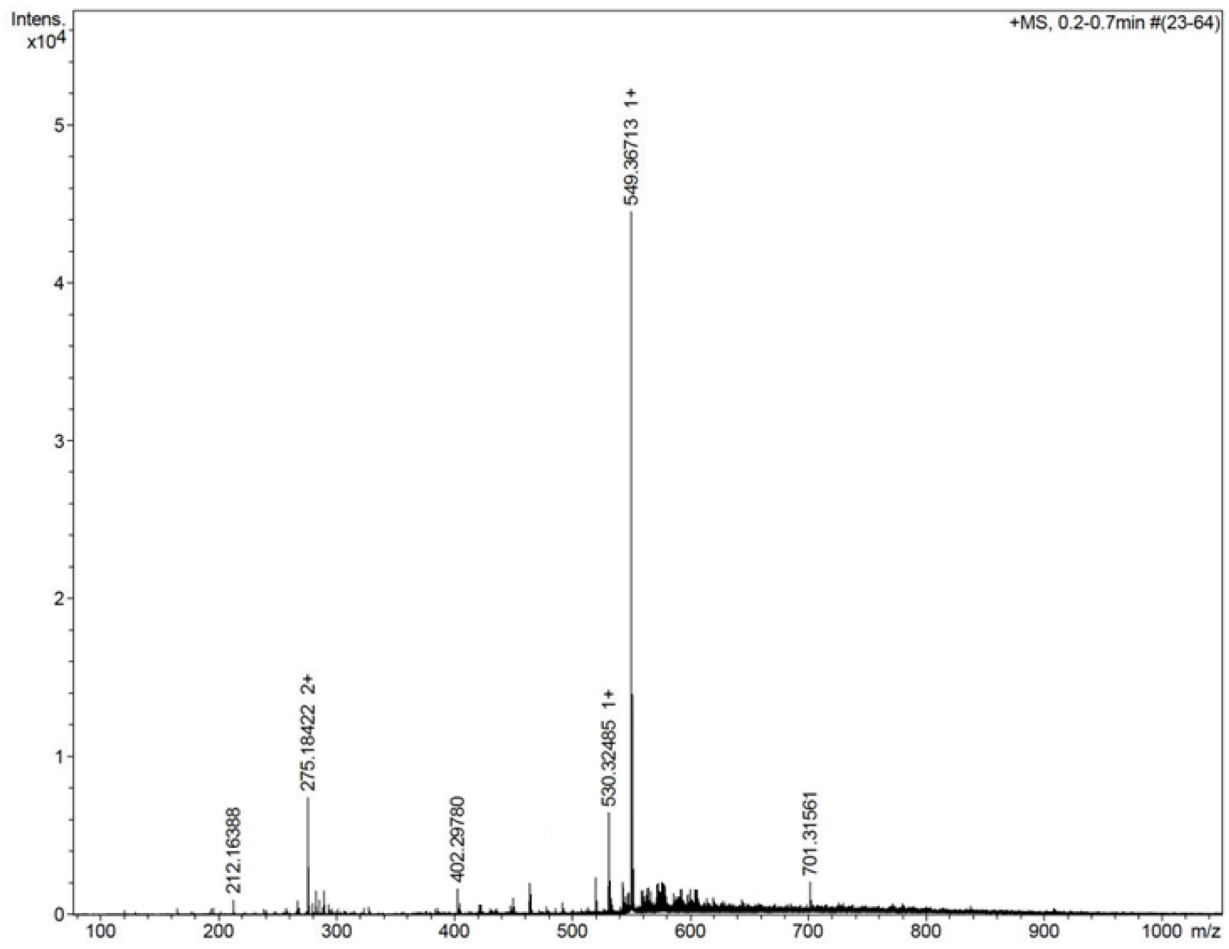
Mass spectrum of G1-dendron showing the main peak of 549.3 representing the exact MW of product with a peak seen at 275.1 with double charge at 0.75*10^4^ intensity, represents the related ion of m/z = (549+1.008*2)/2.

**Figure 5:**
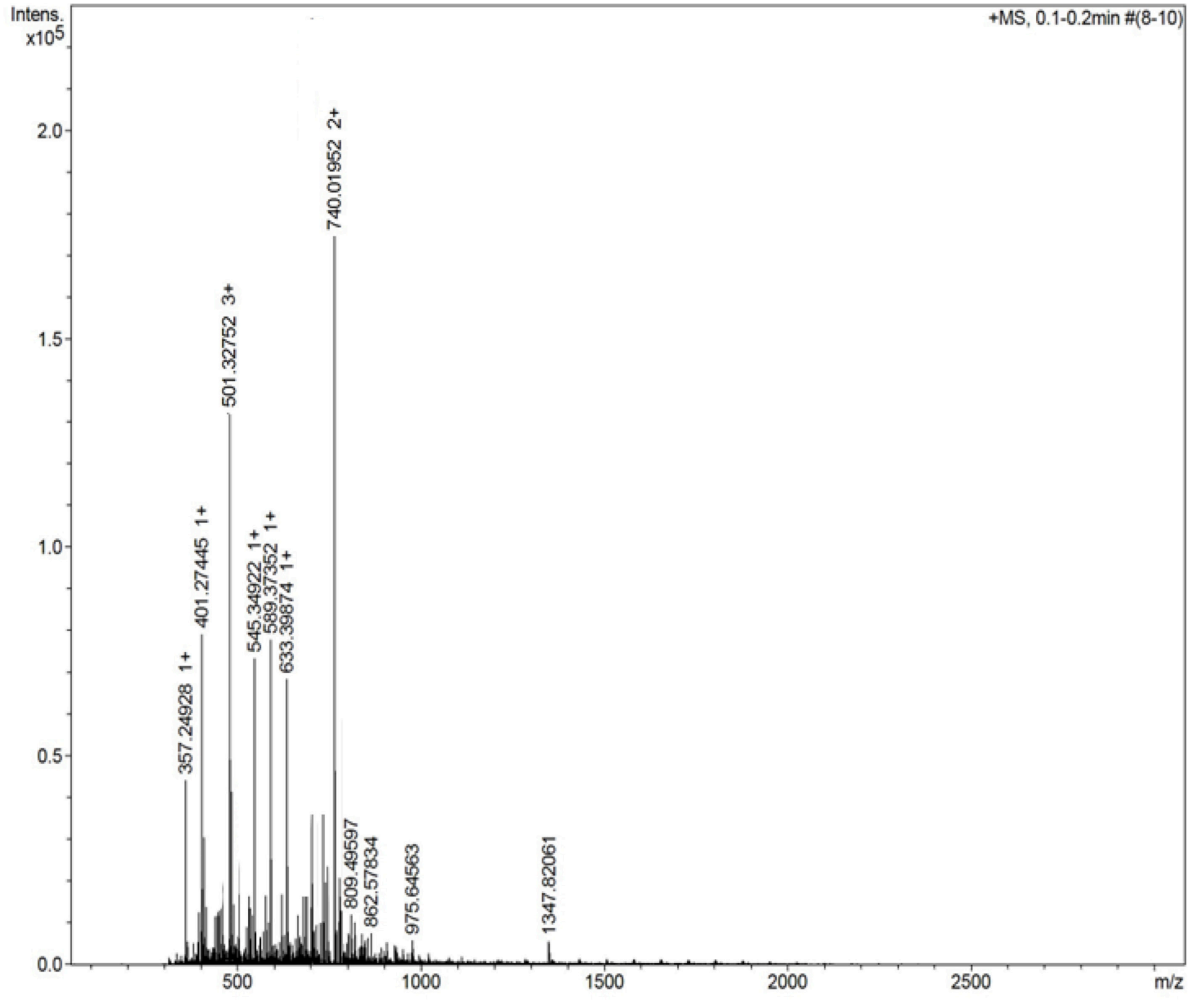
Mass spectrum of AEP-dendron shows a peak seen at 740 with double charge at 1.75*10^5^ intensity, representing the related ion of m/z = (1,479+1.008*2)/2, whereas the peak 501with triple positive charge of intensity 1.3*10^5^ refers to the theoretical MW (m/z = (1,479+1.008*3)/3).

Figure 3 shows the mass spectrum obtained for the AMT-competent G0-dendron, the main peak at 293.2 Da matches exactly the calculated theoretical MW of this molecule indicating the successful synthesis of this dendron. Similarly, Figure 4 refers to the MW of the AMT-competent G1-dendron to be 549.3 Da which again matches the calculated MW of this molecule. The mass spectrum of the RMT-competent AEP-dendron presented in Figure 5 demonstrates the successful synthesis and functionalization of the dendronised peptide with a theoretical MW of 1,479 Da. The appearance of the peak 740 with double charge, and the peak 501 with a triple positive charge, represent the related ions of the AEP-dendron. Other peaks appearing in the spectra result from the ionization of the molecule and the solvent or machine noise, as well as, the related sodium salts formed due to interaction of ions with glass vessels (Downard, 2004). However, the expected molecular weights related to the peptides were clearly observed confirming their synthesis.

### Characterization by HPLC

**Figure 6:**
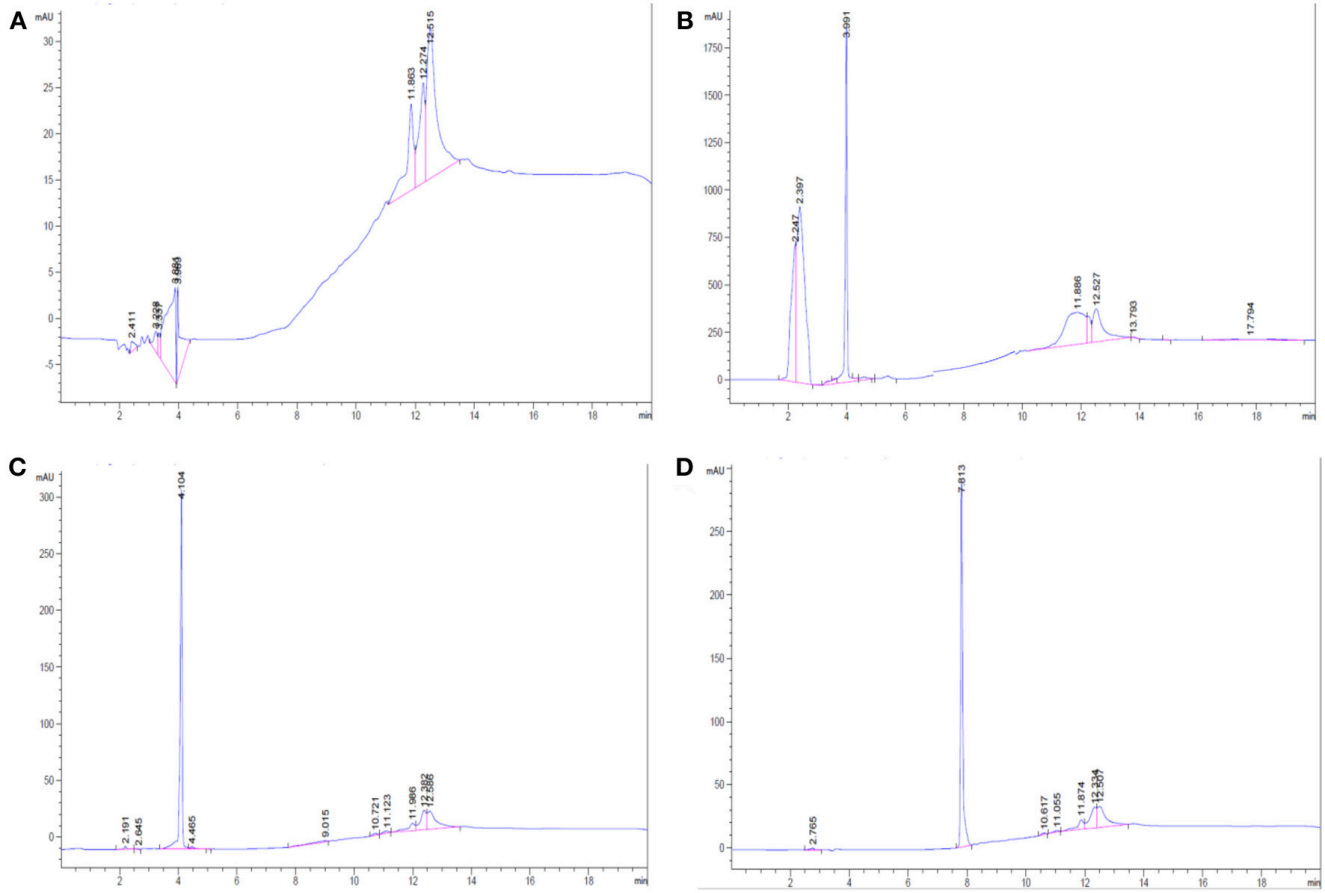
HPLC analysis. **(A)** For Methanol only, **(B)** For G0-dendron in methanol. **(C)** For G1-dendron in methanol and **(D)** For AEP-dendron in methanol.

The synthesized products including the AMT-competent G0-, G1-dendrons, and the RMT-competent AEP-dendron as well as the solvent (methanol) were analyzed by HPLC to characterize the products. It can be noticed that only one large peak was observed in each elution (Figure 6), other small peaks are attributed to the solvent as shown in the elution of methanol alone (Figure 6A). The absence of impurities throughout the HPLC analysis suggested the complete synthesis of the product and cleavage of the linker and resin from the peptide.

### Cytotoxicity Studies

**Figure 7:**
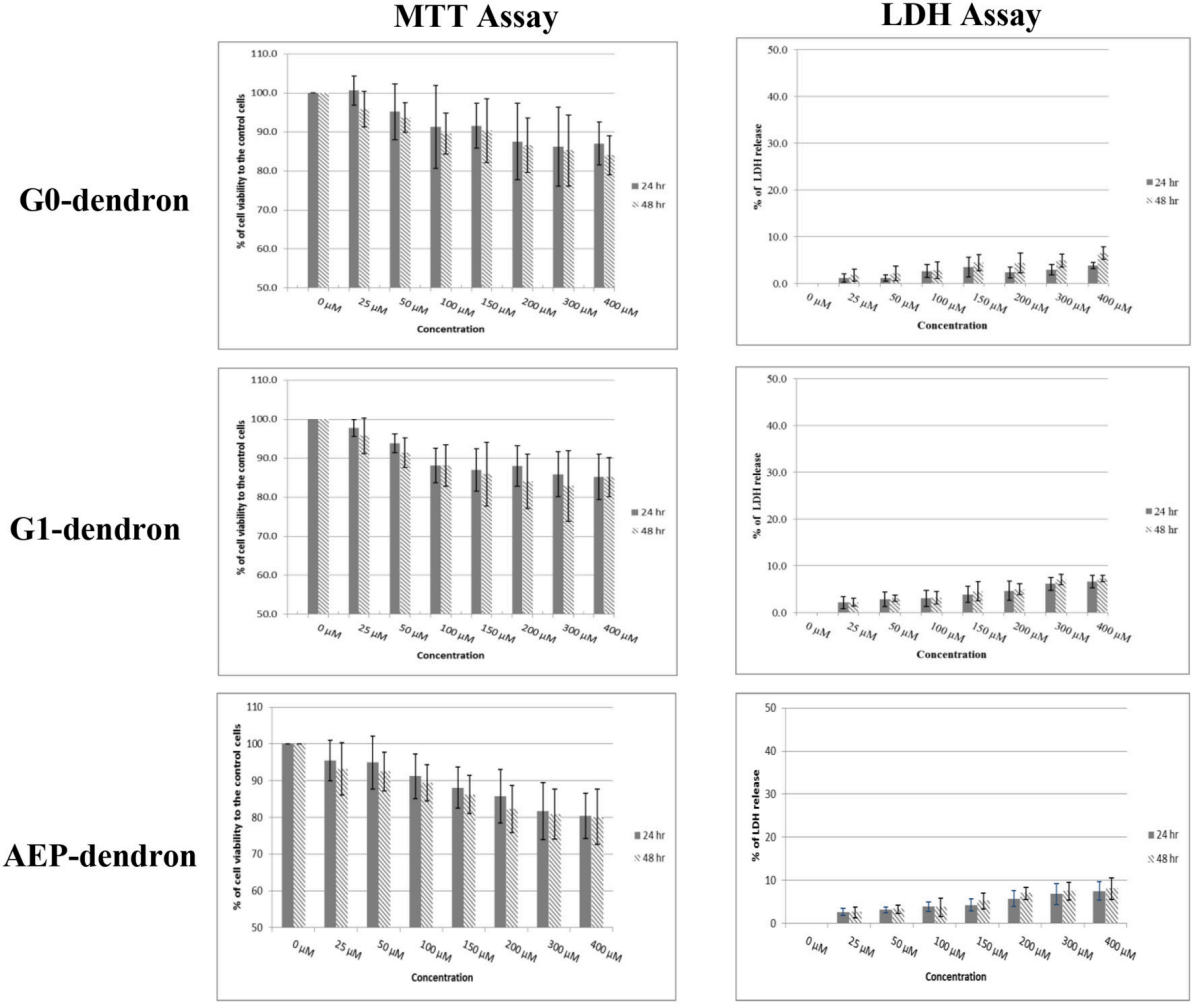
Cytotoxicity results after 24 and 48 h treatment of bEnd.3 cells with G0- and G1-dendron and AEP-dendron. The MTT levels were obtained by measuring absorbance and the cell viability was calculated as a percentage in relation to control untreated cells. The LDH release of each was calculated as a percentage of absorbance in relation to untreated, complete lysis control cells (100% lysis) with significantly difference to positive control (P < 0.001). The data represent mean ± SD of *n* = 6.

The MTT assay was performed after 24 and 48 h treatment of confluent bEnd.3 cells with increasing concentrations of AMT-competent G0- and G1-dendrons, and of RMT-competent AEP-dendron. The results demonstrated no decrease in cell metabolic activity below 78% when compared to the control (Figure 7) suggesting that neither molecules, even at the highest concentration tested (400 μM) were toxic to these cells (International-Standards, 2009). In addition, no significant difference (*P* > 0.05) was seen between the corresponding concentrations after 24 and 48 h treatments.

The results of the LDH assay supported the findings of the MTT assay with low amounts of LDH released after bEnd.3 cells were treated with the AMT-competent G0- and G1-dendrons and with the RMT-competent AEP-dendron in concentrations up to 400 μM for 24 and 48 h. For both incubation periods the cell membrane lysis was below the 10% of the positive control where cells were deliberately lysed with no significant difference (*P* > 0.05) between the corresponding concentrations of both periods (Figure 7).

### Cellular Uptake by Brain Endothelium

#### Cellular Uptake Examination of Dendronised Delivery Systems by LSCM

**Figure 8:**
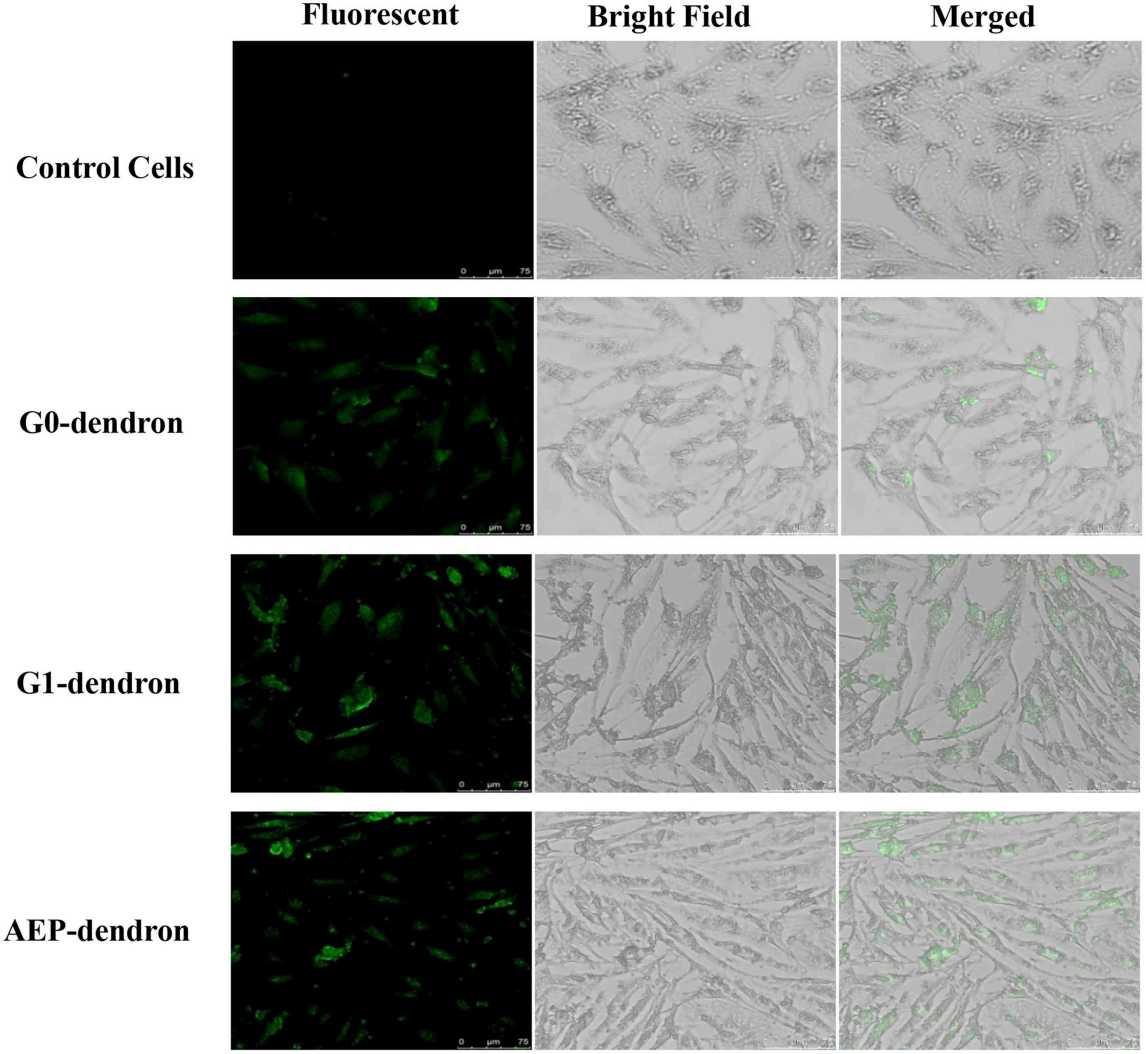
LSCM pictures of bEnd.3 cells after treatment with FITC-labeled G0-, G1-dendron and AEP-dendron and untreated control cells, showing clear accumulation of green fluorescence inside the cells.

After 1 h treatment with FITC-labeled AMT-competent G0- and G1-dendrons and RMT-competent AEP-dendron, the formalin-fixed bEnd.3 cells were viewed by LSCM using a 488-visible laser source. The micrographs clearly showed the accumulation of green fluorescence inside the cells in comparison to the control cells indicating successful cellular uptake of both AMT-competent G0- and G1-dendrons and RMT-competent AEP-dendron (Figure 8). The effect of FITC on the permeability of the delivery systems was excluded by giving negative results when incubated alone with cells (data not shown).

#### Assessment of the Cellular Uptake of Dendronised Delivery Systems by Flow Cytometry

**Figure 9:**
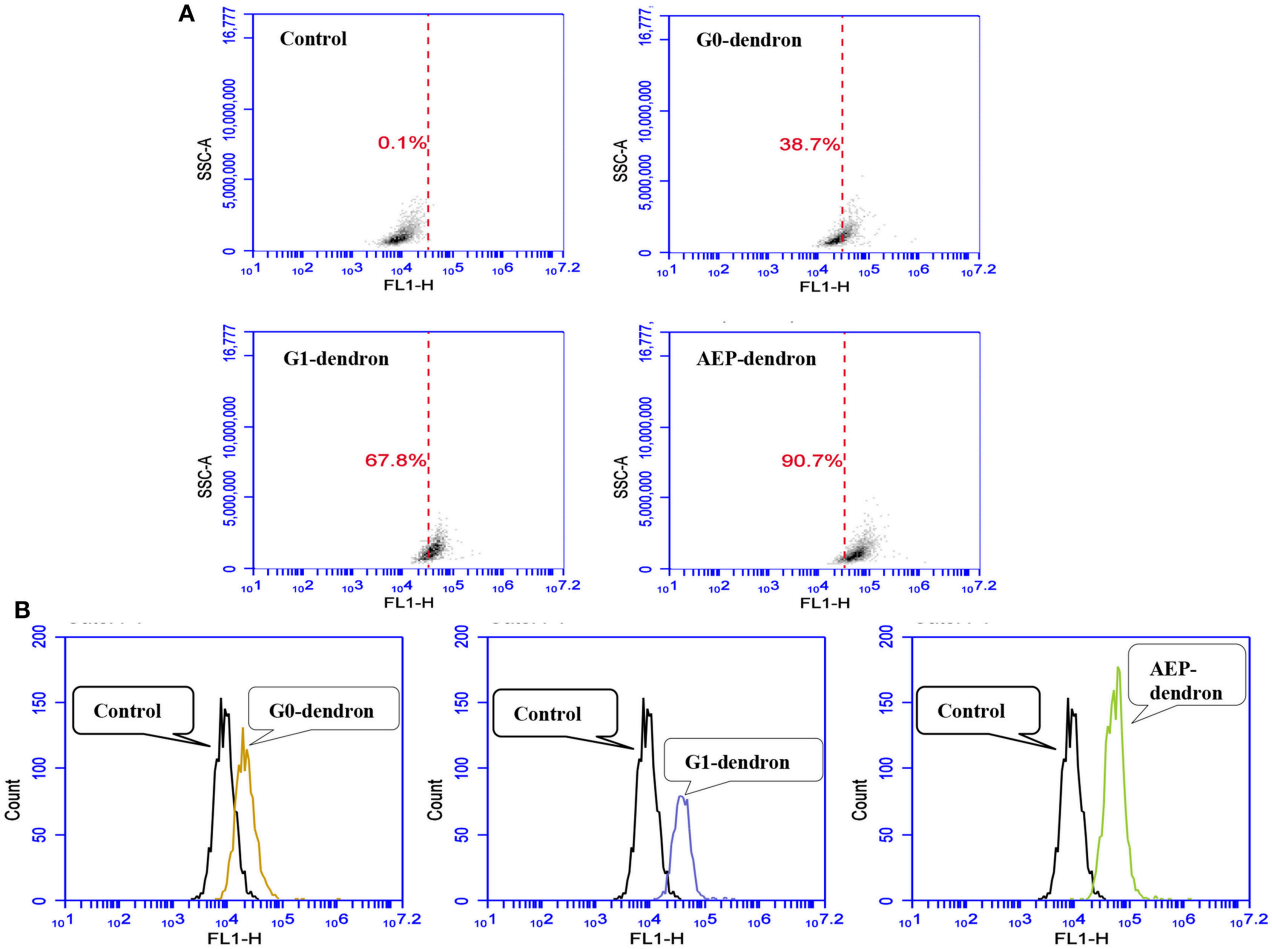
Analysis of FITC-labeled G0-, G1-dendron, and AEP-dendron by flow cytometry through FL1-H channel in comparison to negative control cells. The density blots **(A)** show the uptake percentage of each molecule in comparison to the control. The single-parameter counting histograms **(B)** show clear peak shifting from that of the control.

The uptake of FITC-labeled AMT-competent G0- and G1-dendrons and RMT-competent AEP-dendron was quantitatively analyzed via flow cytometry (Figures 9A,B). The results at 1 h incubation of the carriers with confluent bEnd.3 cells revealed that cellular uptake was 38.7% and 67.8% for the AMT-competent G0- and G1-dendrons in comparison to the negative control (untreated cells). On the other hand, the cellular uptake of the RMT-competent AEP-functionalized dendron reached 90.7% (Figure 9A). In addition, the analysis showed a clear shift of the peaks associated with the AMT-competent G0- and G1-dendrons, and RMT-competent AEP-dendron from that of control untreated cells, with largest shift observed in the RMT-competent AEP-dendron (Figure 9B). The greater the shift from the control the greater is the cellular uptake of the molecule.

## Discussion

The effective and targeted delivery of therapeutics to the brain in NDs remains a huge unsolved problem (Wyss, 2016). As many therapeutics are unable to permeate in to the brain endothelium, several strategies to improve the delivery of molecules including drugs, genes and imaging agents to the CNS have been developed including local injection or BBB opening or enhancing the permeability across the barrier through targeted delivery (Boer and Gaillard, 2007).

In recent years, therapeutic delivery to the brain in NDs has focused on mechanisms that can use the endogenous transport systems available on the BBB which are considered an effective and safe way to deliver molecules into the brain. Indeed, certain peptides and some macromolecules can pass through BBB via transcytosis mechanisms either by physical adsorption on the cell membrane or by receptor mediation (Oller et al., 2016; Mager et al., 2017). Generally, endocytosis in AMT is promoted by the interaction of cationic molecules with phospholipids and the glycocalyx at membrane forming vesicles which matures to early endosomes and the molecule can subsequently be degraded releasing the cargo. Whereas, internalization by RMT is evoked by ligand-receptor interaction, which begins with the formation of a caveolae followed by the delivery of the receptor-ligand complex and transcytosis. In this regards, LDLrs have been extensively exploited in receptor-mediated delivery and signaling at the BBB (Oller et al., 2016). Upregulation of the LDLr gene family at the BBB region, in comparison with other endothelia, can support this hypothesis through the recognition of Apo-E derived peptide (141–150 amino acids) (Re et al., 2011; Wang et al., 2013).

Currently, dendrons offer an important non-invasive strategy for drug delivery and targeting, arising from their structural characteristics with the possibility of multi-functionalisation. In the present study, positively-charged poly(epsilon-Lysine) dendrons (G0 and G1) were used as a carrier system with the potential to be efficiently functionalized at their molecular root with a specific functionality promoting either AMT or RMT, while their free terminal amine groups offer multiple attachments for drug molecules or other bioactive agents. Unlike other approaches, here the size of the carrier and its branching was limited to either one Lysine residue yielding two amino-terminal branches or to two Lysine residues leading to the exposure of four amino-terminals. This approach aimed to preserve the internalization potential of dendrons while reducing the potential toxicity generated by excessive positive charges. In addition, the presence of the hydrophobic domain is deemed to be still beneficial in the case of neutralization of the positive charge of the dendron by interaction with the loaded drug. Indeed, this level of branching is sufficient to support the transport of non-steroideal anti-inflammatory drug across an *in vitro* BBB model using a cultured bEnd.3 monolayer on Transwell membrane (Al-Azzawi et al., 2018).

To achieve this aim, microwave Fmoc-based SPPS was employed to synthesize dendrons and the AEP followed by the functionalization of the dendron at its root with this synthesized AEP (141-150) corresponding to the ApoE-binding domain with LDLr (Re et al., 2011). This procedure ensures a high yield and purity of the final product (Rodriguez et al., 2010). The type of α-amino protecting group in amino acids has to be taken into consideration to ensure the synthesis of the desired peptide. For this reason, Fmoc-Lys(Boc)-OH, was used to obtain a linear chain of AEP, whereas Fmoc-Lys(Fmoc)-OH was employed for the branching of the peptide into a dendron structure. The formation of the amide linkage is dependent upon the side-chain protectors of amino acids which can ensure the chemical reaction of Fmoc at the site of interest only. Therefore, using the appropriate Fmoc type prevents unwanted reactions that could result in the formation or incorporation of dipeptide derivatives. Subsequently, it can help in the purification of the final product due to a smaller amount of secondary products (Made et al., 2014). In this work this was confirmed by the mass spectrometry and HPLC analysis data which indicated the successful synthesis of the desired peptides, matching previous studies in which G3K-dendrimers were efficiently produced and successfully functionalized with different bioactives using this method (Meikle et al., 2011, 2016).

The success of any delivery system or biomaterial to be used in drug or gene delivery relies on their cytocompatibility and biodegradability properties (Svenson and Tomalia, 2005). Based on MTT assay, up to the relatively high doses of 400 μM of AMT-competent G0-, G1-dendrons, and RMT-competent AEP-dendron maintained cell viability after 24 and 48 h above 70% indicating no considerable effect of these novel carriers on mitochondrial function when compared to control cells. Furthermore, the products did not show any significant effect on cell membrane integrity according to the cell lysis results obtained by LDH assays. These findings indicate the biocompatibility of these synthesized products and are consistent with other studies which have shown that low generation dendrons and AEP-functionalized nanocarriers display low cytotoxicity (Sauer et al., 2005). It has been shown that lysine-modified dendrimers demonstrated low cytotoxicity with high transfection efficiency on HepG2, Neuro 2A cell lines, and primary rat vascular smooth muscle cells (Choi et al., 2004). Furthermore, conducting the MTT assay with a hepatocellular carcinoma cell line, have indicated the low cytotoxicity of poly(L-lysine) dendrimer (Li et al., 2007). The lower MTT levels and higher LDH values were observed only with the relatively high concentrations unlikely to be used in clinical applications. Even at such high concentrations the toxicity remained within ranges accepted by the international standards [International-Standards, 2009]. The data also revealed no significant difference (*P* > 0.05) between 2 time intervals incubation which is inconsistent with other studies that have shown the cytotoxicity of dendrons is time dependent (Duncan and Izzo, 2005). A previous study has provided a comprehensive *in vitro* assessment of hydroxyl functional bis-MPA and cationic PAMAM dendrimers and revealed that cytotoxicity is increasing with time (Feliu et al., 2012). The type of cell line and the dendron compositions could be the reasons beyond these controversial observations. The results in this study suggest that molecules could be internalized over a relatively short period of time (1 h *in vitro*) and without harmful effect to the BBB endothelial cells.

Cellular uptake by the BBB endothelial cells was confirmed by LSCM analysis of FITC-labeled carriers. The cellular binding and uptake of the AMT-competent G0 and G1 dendrons were shown. It has been suggested that dendrimers can successfully be internalized by cells, possibly via adsorptive endocytosis here facilitated by the combination of the overall positive charge of the poly(epsilon-Lysine) dendron and the presence of a relatively hydrophobic phenylalanine at the root of the carrier (Kolhe et al., 2003; Patri and Simanek, 2012; Sadekar et al., 2013). After bEnd.3 cells were incubated with the RMT-competent AEP-dendron, a more evident intracellular uptake of the FITC-labeled carrier could be observed in comparison to the control and to the AMT-competent carriers. These results supported the hypothesis of a more efficient, receptor-mediated uptake of the AEP-decorated dendron via the lipoprotein receptor. These findings were in agreement with other studies which showed that nanoparticles decorated with AEP were internalized by bEnd.3 cell line via interaction with LDLr (Zensi et al., 2009; Wagner et al., 2012).

The findings obtained by LSCM, provided only a qualitative assessment of the intracellular uptake of the carriers and required a quantitative evaluation by flow cytometry. This study allowed to assess the effect of the size and biospecificity of the carrier on cell internalization. Noticeably, the uptake of the AMT-competent G1-dendron was nearly the double of that measured in the case of the G0-dendron, suggesting that the increased number of amino branches and the higher number of positive charges on G1 played a role more relevant than the hydrophobic domain present at the root of the dendron. Indeed, it has previously been shown that the cellular entry of G4-dendrimer was higher than G3-dendrimer (Najlah and D'Emanuele, 2006) and the cationic PAMAM dendrimers (G0-G4) permeability has been found to increase with generation owing to a greater number of peripheral positively-charged amino groups (Kitchens et al., 2005). However, a significantly higher uptake of carriers by the bEnd.3 cells was observed when a G0 dendron was root-modified with the AEP-mimicking peptide. This suggests that this carrier system entered the bEnd.3 cells, most likely by a receptor-mediated pathway. This speculation is supported by previous studies that have assessed the ApoE derived peptides intracellular distribution that showed that their uptake is mediated by receptor endocytosis (Re et al., 2010; Bana et al., 2014). The binding domain of the ApoE residue (141–150) has previously been found to be able to induce a cellular uptake via LDLr- engagement in primary cell cultures of brain tissue (X. Wang et al., 1997). Furthermore, nanoliposomes decorated with the ApoE-derived peptide have been shown to be efficiently internalized by brain endothelium of rat after 30 min incubation when compared to free nanoliposome (Sauer et al., 2005). In addition, nanoliposomes decorated with the ApoE-derived peptide (141–150) have been shown to successfully enhance the brain uptake of antioxidants (Gobbi et al., 2010) and curcumin (Re et al., 2011). A further study has shown a specific cellular binding after incubation of bEnd.3 cells with nanoparticles covalently linked to ApoE in comparison to the unlinked nanoparticles using confocal microscopy and flow cytometry analysis (Zensi et al., 2009; Wagner et al., 2012).

This study has demonstrated that dendronised carriers are able to be internalized by brain endothelia, and, to a greater extent, when functionalized with AEP according to an RMT strategy. This internalization is considered the initial step for transporting bioactive materials into the brain. An attractive aspect of the RMT strategy for CNS drug delivery is that it targets specific organs, in turn, it bypasses, or reduced the uptake by, other body tissues decreasing any undesirable systemic effects. This is unlike other approaches which lack selective brain targeting leading to higher distribution in the systemic circulation and inadequate amounts delivered to the brain (Jones and Shusta, 2007; Mager et al., 2017). Alternative entry routes such as direct injection into the cerebrospinal fluid or temporary opening to the BBB are limited by the requirement of hospitalization, invasiveness and increased infection risk and pathogen entry to the brain (Chen and Liu, 2012).

Peptides including AEP, are affordable, easily obtained and characterized with their suitability to chemical modification which opens up the possibility of applying them to a variety of strategies where multiple functional groups for site-specific conjugation can be added (Oller et al., 2016). Furthermore, in the case of RMT, most peptide ligands neither compete with endogenous compounds nor stay bound to the receptor when compared to some antibodies that have been previously used as carriers (Xiao and Gan, 2013; Oller et al., 2016). Thus receptor-based BBB transporters have so far provided promising achievements in preclinical brain delivery (Mager et al., 2017; Molino et al., 2017).

Taking all the currently available evidence together, these dendronised carrier systems produced using a solid-phase synthesis optimized method have the potential to provide a novel platform of BBB delivery systems for drugs (e.g., Flurbiprofen in Alzheimer's as previously shown, Al-Azzawi et al., 2018) or genes that are currently ineffective or unavailable to the prescriber due to their poor penetration of the BBB. Moreover, these dendronised delivery systems are not only non-toxic but also offers the potential to improve the drug's bioavailability due to their capability to couple large amounts of drug molecules to their branching ends.

## Author Contributions

SA and DM performed the experiments and wrote the manuscript. AG, GP, and MS supervised the work, analyzed the data and edited the manuscript. MS planned and supervised the whole study.

### Conflict of Interest Statement

The authors declare that the research was conducted in the absence of any commercial or financial relationships that could be construed as a potential conflict of interest.
